# Supplementary material for: Distinct changes to hippocampal and medial entorhinal circuits emerge across the progression of cognitive deficits in epilepsy
Source: bioRxiv. 2024 Mar 14:2024.03.12.584697. Preprint. [Version 1] doi: 10.1101/2024.03.12.584697 (PMC10979962; doi:10.1101/2024.03.12.584697)
Supplement: Supplement 1 [file NIHPP2024.03.12.584697v1-supplement-1.pdf]

bioRxiv preprint doi: https://doi.org/10.1101/2024.03.12.584697; this version posted March 14, 2024. The copyright holder for this preprint (which was not certified by peer review) is the author/funder, who has granted bioRxiv a license to display the preprint in perpetuity. It is made available under aCC-BY 4.0 International license.

SUPPLEMENTARY FIGURES

Supplementary Figure 1

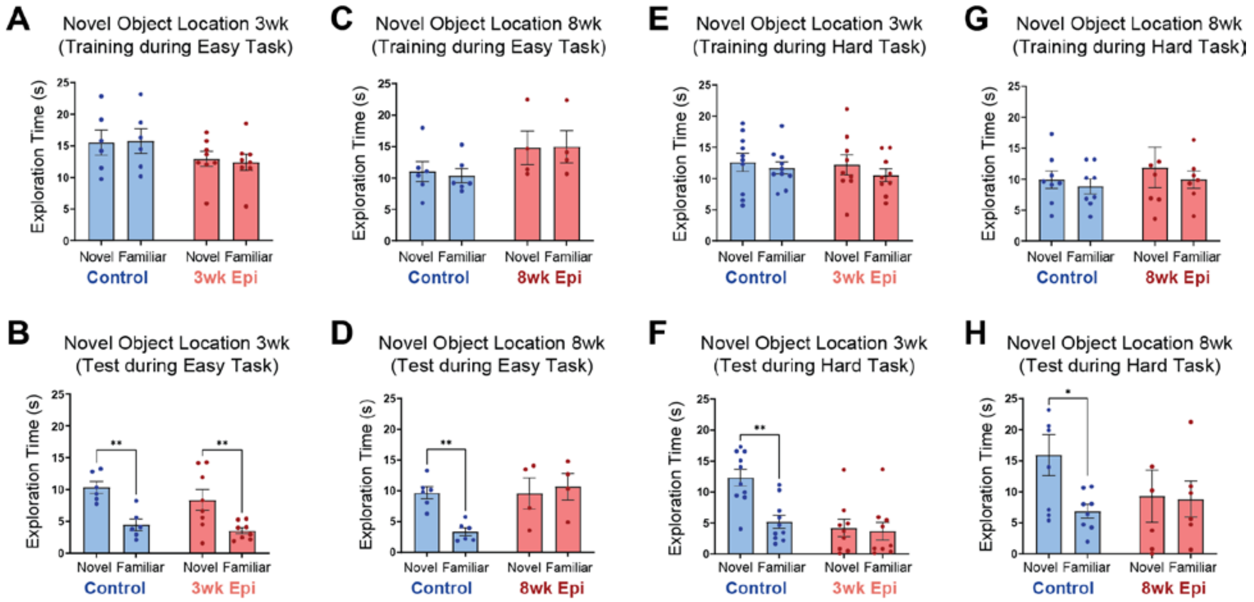

Supplementary Figure 1: Novel object location (NOL) exploration times during training and testing
A. No differences in exploration time between the two objects in either Control or 3wk Epileptic groups during training on the Easy NOL task (N = 6, Control; N = 8, 3wk Epileptic; 2-way repeated measures ANOVA, $F_{object}(1,12) = 0.2$, $p > 0.05$).
B. Both Control and 3wk Epileptic groups spent more time with the moved object during testing on the Easy NOL task (N = 6, Control; N = 8, 3wk Epileptic; 2-way repeated measures ANOVA, $F_{object}(1,12) = 24.9$, $p < 0.001$; Bonferroni corrected: Control: Novel v. Familiar $p < 0.01$; 3wk Epileptic: Novel v. Familiar $p < 0.01$).
C. No differences in exploration time between the two objects in either Control or 8wk Epileptic groups during training on the Easy NOL task (N = 6, Control; N = 4, 8wk Epileptic; 2-way repeated measures ANOVA, $F_{object}(1,8) = 0.2$, $p > 0.05$).
D. During testing on the Easy NOL task, the Control group spent more time with the moved object while the 8wk Epileptic group spent equal time with both objects (N = 6, Control; N = 4, 8wk Epileptic; 2-way repeated measures ANOVA, $F_{object}(1,8) = 8$, $p < 0.05$; Bonferroni corrected: Control: Novel v. Familiar $p < 0.01$; 8wk Epileptic: Novel v. Familiar $p > 0.05$).
E. No differences in exploration time between the two objects in either Control or 3wk Epileptic group during training on the Hard NOL task (N = 10, Control; N = 9, 3wk Epileptic; 2-way repeated measure ANOVA, $F_{object}(1,17) = 2.5$, $p > 0.05$).
F. During testing on the Hard NOL task, the Control group spent more time with the moved object while the 3wk Epileptic group spent equal time with both objects (N = 10, Control; N = 9, 3wk Epileptic; 2-way repeated measure ANOVA, $F_{object}(1,17) = 8.5$ $p < 0.01$; Bonferroni corrected: Control: Novel v. Familiar $p < 0.01$; 3wk Epileptic: Novel v. Familiar $p > 0.05$).
G. No differences in exploration time between the two objects in either Control or 8wk Epileptic group during training on the Hard NOL task (N = 8, Control; N = 7, 8wk Epileptic; 2-way repeated measure ANOVA, $F_{object}(1,13) = 1.5$, $p > 0.05$).
H. During testing on the Hard NOL task, the Control group spent more time with the moved object while the 8wk Epileptic group spent equal time with both objects (N = 8, Control; N = 6, 8wk Epileptic; 2-way repeated measure ANOVA, $F_{object}(1,12) = 3.4$, $p = 0.09$; Bonferroni corrected: Control: Novel v. Familiar $p < 0.05$; 8wk Epileptic: Novel v. Familiar $p > 0.05$).
Error bars represent s.e.m. *p<0.05, **p<0.01, ***p<0.001

bioRxiv preprint doi: https://doi.org/10.1101/2024.03.12.584697; this version posted March 14, 2024. The copyright holder for this preprint (which was not certified by peer review) is the author/funder, who has granted bioRxiv a license to display the preprint in perpetuity. It is made available under aCC-BY 4.0 International license.

Supplementary Figure 2

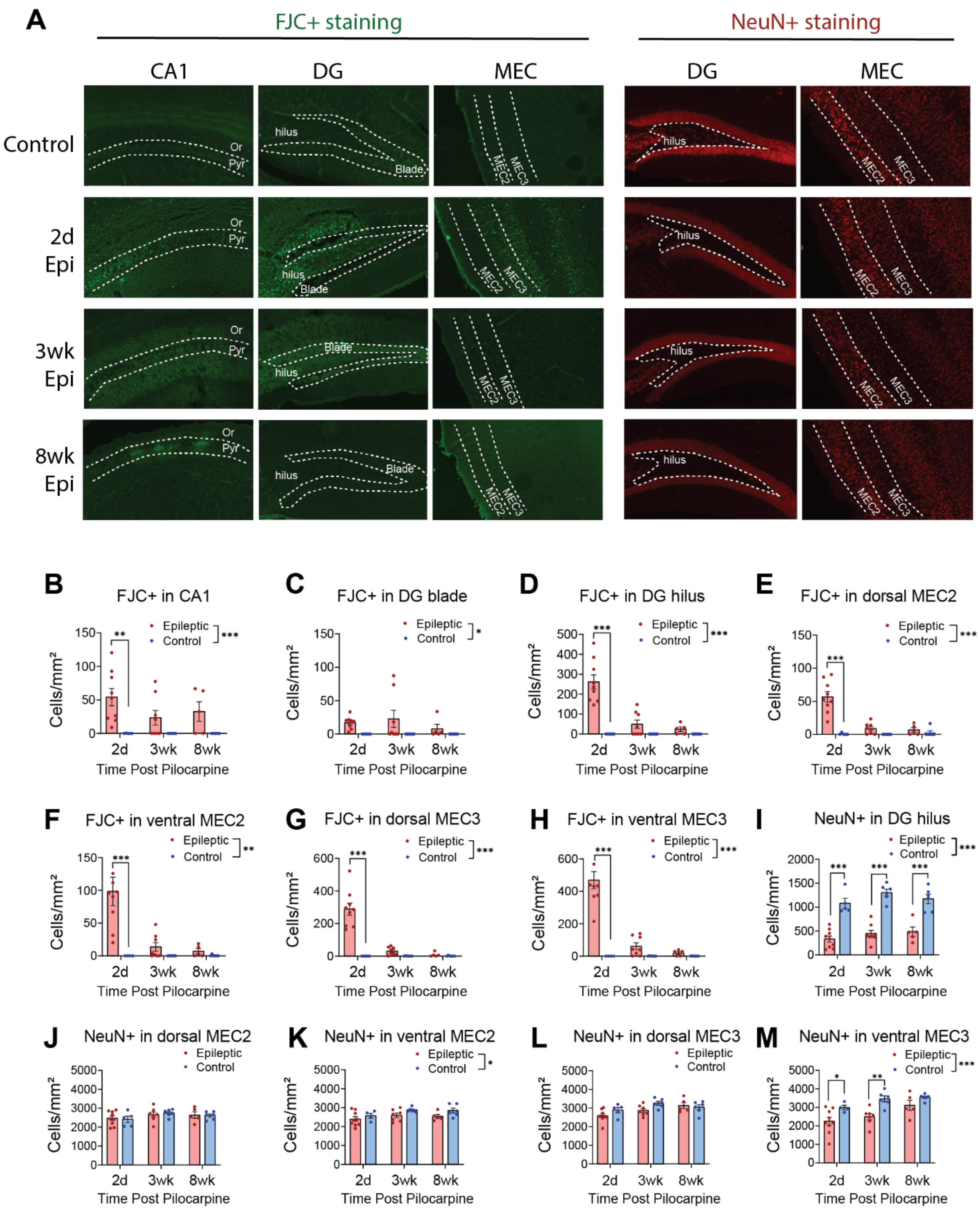

bioRxiv preprint doi: https://doi.org/10.1101/2024.03.12.584697; this version posted March 14, 2024. The copyright holder for this preprint (which was not certified by peer review) is the author/funder, who has granted bioRxiv a license to display the preprint in perpetuity. It is made available under aCC-BY 4.0 International license.

Supplementary Figure 2: Neurodegenerative signaling and cell loss at 2 days, 3 weeks, and 8 weeks after Pilo-SE

A. Example immunohistochemistry staining for Fluro Jade-C (FJC, left) in CA1, DG (hilus and blade), MEC (MEC2 and MEC3), and NeuN (right) in DG (hilus) and MEC (MEC2 and MEC3) of Control and Epileptic mice. For Epileptic mice, tissue was collected at 2 days, 3 weeks, and 8 weeks after Pilo-SE.

B. FJC staining was increased in CA1 in 2d Epileptic group, with group differences between Control and Epileptic groups (N = 5, 2d Control; N = 6, 3wk Control; N = 6, 8wk Control; N = 9, 2d Epileptic; N = 8, 3wk Epileptic; N = 5, 8wk Epileptic. 2-way ANOVA, $F_{group}(1,33) = 18.2$, $p < 0.001$; Bonferroni corrected: Control v. 2d Epileptic $p < 0.01$).

C. FJC staining shows group level reduction in DG blade in epileptic groups (N = 5, 2d Control; N = 6, 3wk Control; N = 6, 8wk Control; N = 9, 2d Epileptic; N = 8, 3wk Epileptic; N = 5, 8wk Epileptic. 2-way ANOVA, $F_{group}(1,33) = 7.2$, $p < 0.05$).

D. FJC staining was increased in DG hilus in the 2d Epileptic group, with a main effect between Control and Epileptic groups (N = 5, 2d control; N = 6, 3wk Control; N = 6, 8wk Control; N = 9, 2d Epileptic; N = 8, 3wk Epileptic; N = 5, 8wk Epileptic. 2-way ANOVA, $F_{group}(1,33) = 32$, $p < 0.0001$; Bonferroni corrected: Control v. 2d Epileptic $p < 0.0001$).

E. FJC staining was increased in dorsal MEC2 in the 2d Epileptic group, with a main effect between Control and Epileptic groups (N = 5, 2d Control; N = 6, 3wk Control; N = 6, 8wk Control; N = 9, 2d Epileptic; N = 8, 3wk Epileptic; N = 5, 8wk Epileptic. 2-way ANOVA, $F_{group}(1,33) = 29.3$, $p < 0.0001$; Bonferroni corrected: Control v. 2d Epileptic $p < 0.0001$).

F. FJC staining was increased in ventral MEC2 in the 2d Epileptic group, with a main effect between Control and Epileptic groups (N = 5, 2d Control; N = 6, 3wk Control; N = 6, 8wk Control; N = 9, 2d Epileptic; N = 8, 3wk Epileptic; N = 5, 8wk Epileptic. 2-way ANOVA, $F_{group}(1,33) = 12$, $p < 0.01$; Bonferroni corrected: Control v. 2d Epileptic $p < 0.0001$).

G. FJC staining was increased in dorsal MEC3 in 2d Epileptic group, with a main effect between Control and Epileptic groups (N = 5, 2d Control; N = 6, 3wk Control; N = 6, 8wk Control; N = 9, 2d Epileptic; N = 8, 3wk Epileptic; N = 5, 8wk Epileptic. 2-way ANOVA, $F_{group}(1,33) = 34.1$, $p < 0.0001$; Bonferroni corrected: Control v. 2d Epileptic $p < 0.0001$).

H. FJC staining was increased in ventral MEC3 in 2d Epileptic group, with a main effect between Control and Epileptic groups (N = 4, 2d Control; N = 6, 3wk Control; N = 6, 8wk Control; N = 9, 2d Epileptic; N = 8, 3wk Epileptic; N = 5, 8wk Epileptic. 2-way ANOVA, $F_{group}(1,32) = 42.3$, $p < 0.0001$; Bonferroni corrected: Control v. 2d Epileptic $p < 0.0001$).

I. NeuN staining was decreased in DG hilus in all Epileptic groups, with a main effect between Control and Epileptic groups (N = 5, 2d Control; N = 6, 3wk Control; N = 6, 8wk Control; N = 9, 2d Epileptic; N = 8, 3wk Epileptic; N = 5, 8wk Epileptic. 2-way ANOVA, $F_{group}(1,33) = 128.3$, $p < 0.0001$; Bonferroni corrected: Control v. 2d Epileptic $p < 0.0001$; Control v. 3wk Epileptic $p < 0.0001$; Control v. 8wk Epileptic $p < 0.0001$).

J. NeuN staining showed no difference in dorsal MEC2 between Epileptic and Control groups (N = 5, 2d Control; N = 6, 3wk Control; N = 6, 8wk Control; N = 9, 2d Epileptic; N = 7, 3wk Epileptic; N = 5, 8wk Epileptic. 2-way ANOVA, $F_{group}(1,32) = 0$, $p > 0.05$).

K. NeuN staining was reduced in ventral MEC2 with a main effect between Control and Epileptic groups (N = 4, 2d Control; N = 6, 3wk Control; N = 6, 8wk Control; N = 9, 2d Epileptic; N = 7, 3wk Epileptic; N = 5, 8wk Epileptic. 2-way ANOVA, $F_{group}(1,31) = 6.6$, $p < 0.05$).

L. NeuN staining showed no difference in dorsal MEC3 between Epileptic and Control groups (N = 5, 2d Control; N = 6, 3wk Control; N = 6, 8wk Control; N = 9, 2d Epileptic; N = 7, 3wk Epileptic; N = 5, 8wk Epileptic. 2-way ANOVA, $F_{group}(1,32) = 3.7$, $p > 0.05$).

M. NeuN staining was decreased in ventral MEC3 in 2d and 3wk Epileptic groups, with a main effect between Control and Epileptic groups (N = 4, 2d Control; N = 6, 3wk Control; N = 6, 8wk Control; N = 9, 2d Epileptic; N = 8, 3wk Epileptic; N = 5, 8wk Epileptic. 2-way ANOVA, $F_{group}(1,31) = 19$, $p < 0.001$; Bonferroni corrected: Control v. 2d Epileptic $p < 0.05$; Control v. 3wk Epileptic $p < 0.01$).

Error bars represent s.e.m. *p<0.05, **p<0.01, ***p<0.001

bioRxiv preprint doi: https://doi.org/10.1101/2024.03.12.584697; this version posted March 14, 2024. The copyright holder for this preprint (which was not certified by peer review) is the author/funder, who has granted bioRxiv a license to display the preprint in perpetuity. It is made available under aCC-BY 4.0 International license.

Supplementary Figure 3

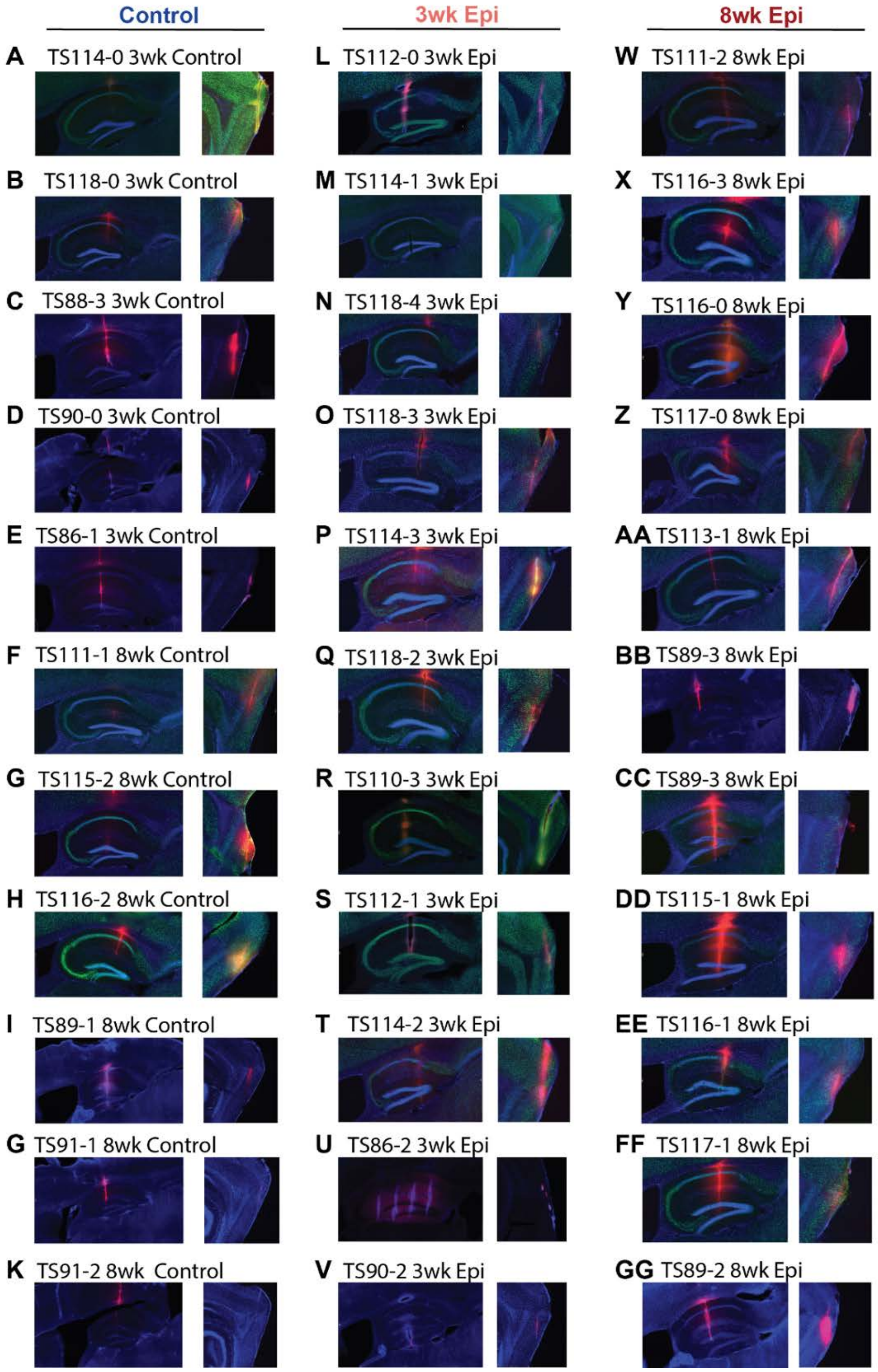

Supplementary Figure 3: Representative probe tract for each animal
A-K. Probe tracts in all Control animals
L-V. Probe tracts in all 3wk Epileptic (Epi) animals
W-GG. Probe tracts in all 8wk Epileptic (Epi) animals
Red: probe tract; Green: NeuN; Blue: DAPI

bioRxiv preprint doi: https://doi.org/10.1101/2024.03.12.584697; this version posted March 14, 2024. The copyright holder for this preprint (which was not certified by peer review) is the author/funder, who has granted bioRxiv a license to display the preprint in perpetuity. It is made available under aCC-BY 4.0 International license.

Supplementary Figure 4

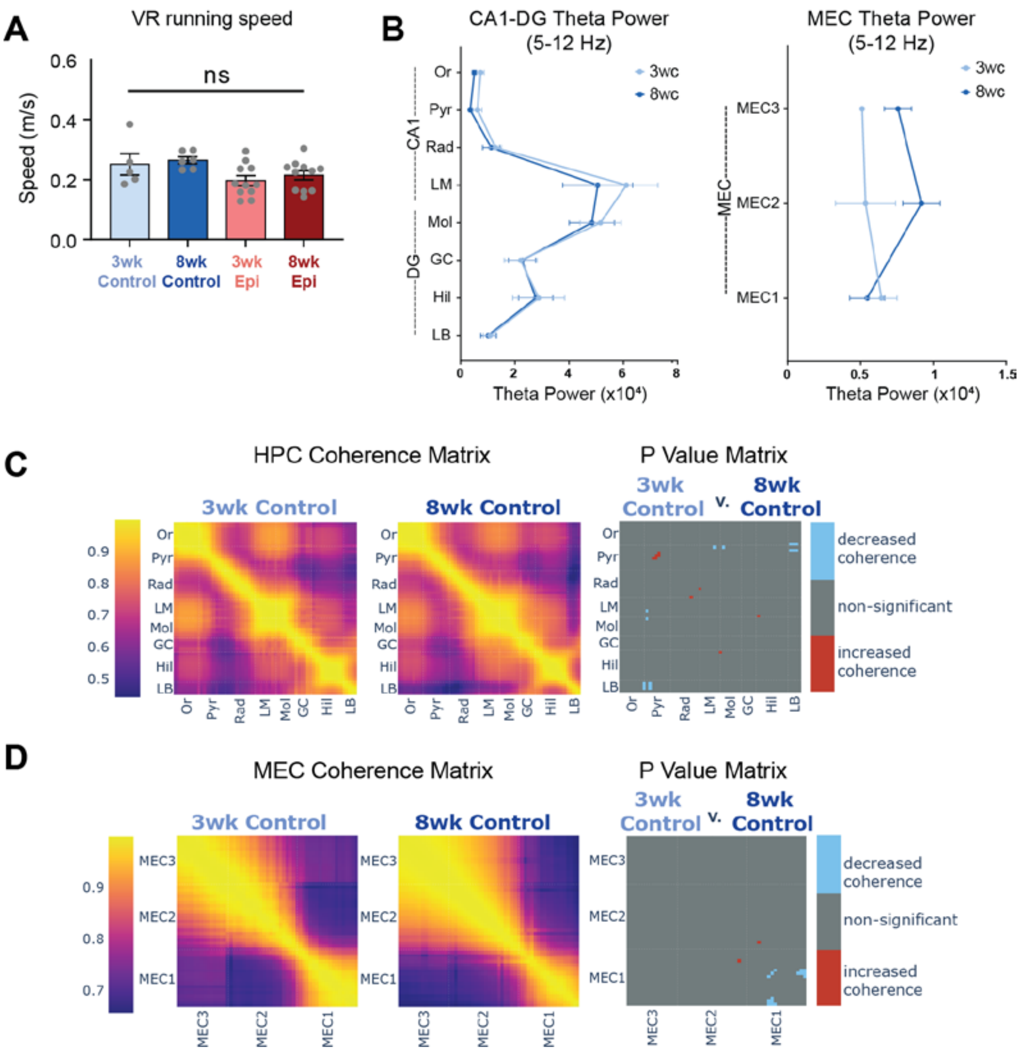

Supplementary Figure 4: No differences in running speed across groups or theta power and coherence in Control animals.
A. No differences in average running speed in VR during recordings (N = 5, 3wk Control; N = 6, 8wk Control; N = 11, 3wk Epileptic; N = 11, 8wk Epileptic. One-way ANOVA, $F(3,29)$ = 2.6, $p > 0.05$).
B. Theta power from each hippocampus layer (left) and MEC layer (right) in 3wk and 8wk Control animals. No differences were detected in any region (N = 5, 3wk Control; N = 6, 8wk Control; For HPC: Repeated-measures mixed-effects model, $F_{Group\ x\ Region}$ (7,61) = 0.2, $p > 0.05$; For MEC: Repeated-measures mixed-effects model, $F_{Group\ x\ Region}$ (2, 12) = 2.3, $p > 0.05$).
C. Theta coherence between each channel pair along the probe in HPC in 3wk Control (left) and 8wk Control (middle) groups. P value matrix (right) shows the significant comparisons from each region pairs in HPC between groups. No clear patterns of significant differences were detected (N = 5, 3wk Control; N = 6, 8wk Control; welch t-test with alpha = 0.05; blue: $p < 0.05$, decrease coherence; red: $p < 0.05$, increase coherence).
D. Theta coherence between each channel pair along the probe in MEC in 3wk Control (left) and 8wk Control (middle) groups. P value matrix (right) shows the significant comparisons from each region pairs in MEC between groups. No clear patterns of significant difference were detected (N = 5, 3wk Control; N = 6, 8wk Control; welch t-test with alpha = 0.05; blue: $p < 0.05$, decrease coherence; red: $p < 0.05$, increase coherence).
Error bars represent s.e.m. *p<0.05, **p<0.01, ***p<0.001

bioRxiv preprint doi: https://doi.org/10.1101/2024.03.12.584697; this version posted March 14, 2024. The copyright holder for this preprint (which was not certified by peer review) is the author/funder, who has granted bioRxiv a license to display the preprint in perpetuity. It is made available under aCC-BY 4.0 International license.

Supplementary Figure 5

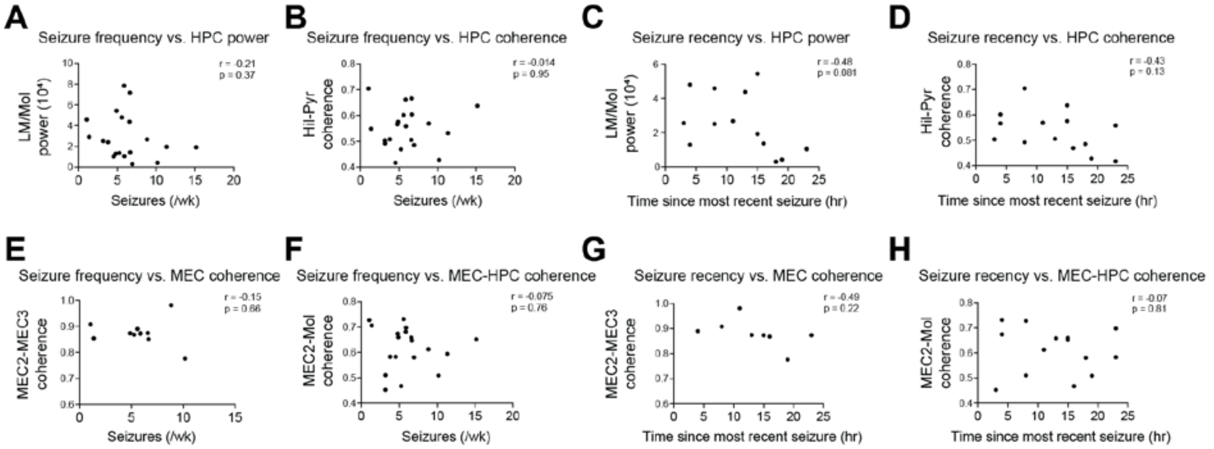

Supplementary Figure 5: Seizure frequency and seizure recency is not correlated with theta power or coherence in Epileptic animals.

A. No correlation between seizure frequency and HPC (LM and Mol layers) theta power (N = 20, Epileptic animals; Pearson r = -0.21, p > 0.05).

B. No correlation between seizure frequency and HPC (Hil and Pyr layers) theta coherence (N = 20, Epileptic animals; Pearson r = -0.014, p > 0.05).

C. No correlation between the time since the most recent seizure and theta power in HPC (LM and Mol layers) (N = 14, Epileptic animals; Pearson r = -0.48, p > 0.05).

D. No correlation between the time since the most recent seizure and theta coherence between Hil and Pyr layers (N = 14, Epileptic animals; Pearson r = -0.43, p > 0.05).

E-H. No correlation between seizure frequency and MEC (MEC2 and MEC3 layers) theta coherence (E: N = 11, Epileptic animals; Pearson r = -0.15, p > 0.05) or seizure frequency and MEC-HPC (MEC2 and Molecular layers) theta coherence (F: N = 19, Epileptic animals; Pearson r = -0.075, p > 0.05). No correlation between the time since the most recent seizure and theta coherence between layers MEC2 and MEC3 (G: N = 8, Epileptic animals; Pearson r = -0.49, p > 0.05) or theta coherence between MEC2 and HPC Molecular layer (H: N = 14, Epileptic animals; Pearson r = -0.07, p > 0.05).

bioRxiv preprint doi: https://doi.org/10.1101/2024.03.12.584697; this version posted March 14, 2024. The copyright holder for this preprint (which was not certified by peer review) is the author/funder, who has granted bioRxiv a license to display the preprint in perpetuity. It is made available under aCC-BY 4.0 International license.

Supplementary Figure 6

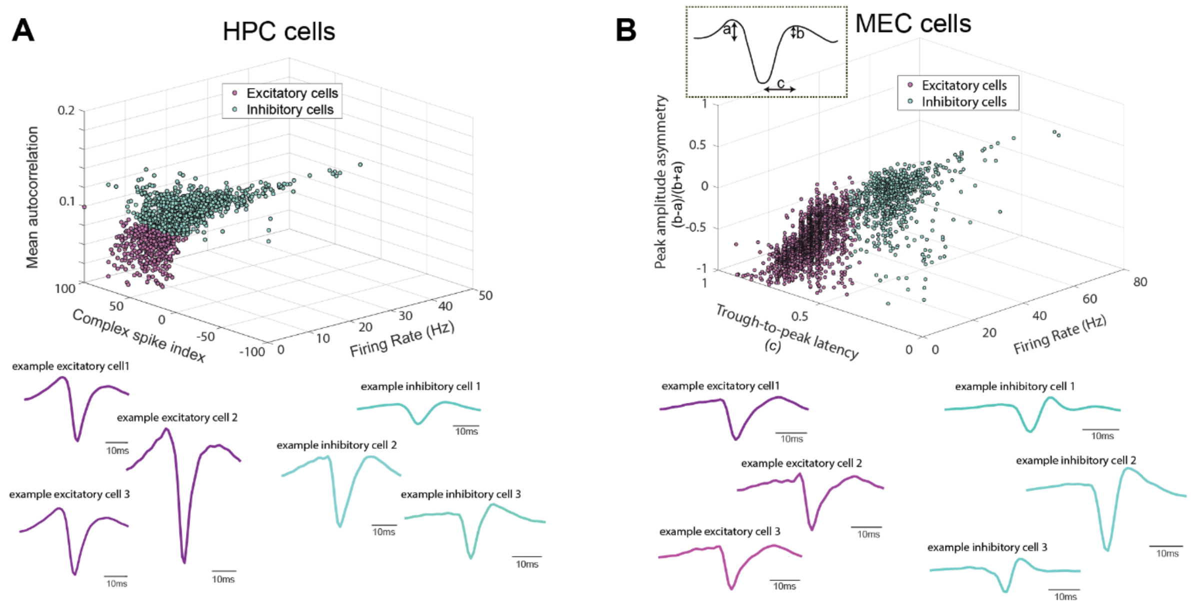

Supplementary Figure 6: HPC and MEC putative excitatory and inhibitory cells
A. Top: Putative excitatory and inhibitory cells in HPC separated by mean autocorrelation, complex spike index, and firing rate. Bottom: 3 examples of excitatory HPC cells and 3 examples of inhibitory HPC cells.
B. Top: Putative excitatory and inhibitory cells in MEC separated by trough-to-peak latency and peak amplitude asymmetry. Bottom: 3 examples of excitatory MEC cells and 3 examples of inhibitory MEC cells

bioRxiv preprint doi: https://doi.org/10.1101/2024.03.12.584697; this version posted March 14, 2024. The copyright holder for this preprint (which was not certified by peer review) is the author/funder, who has granted bioRxiv a license to display the preprint in perpetuity. It is made available under aCC-BY 4.0 International license.

Supplementary figure 7

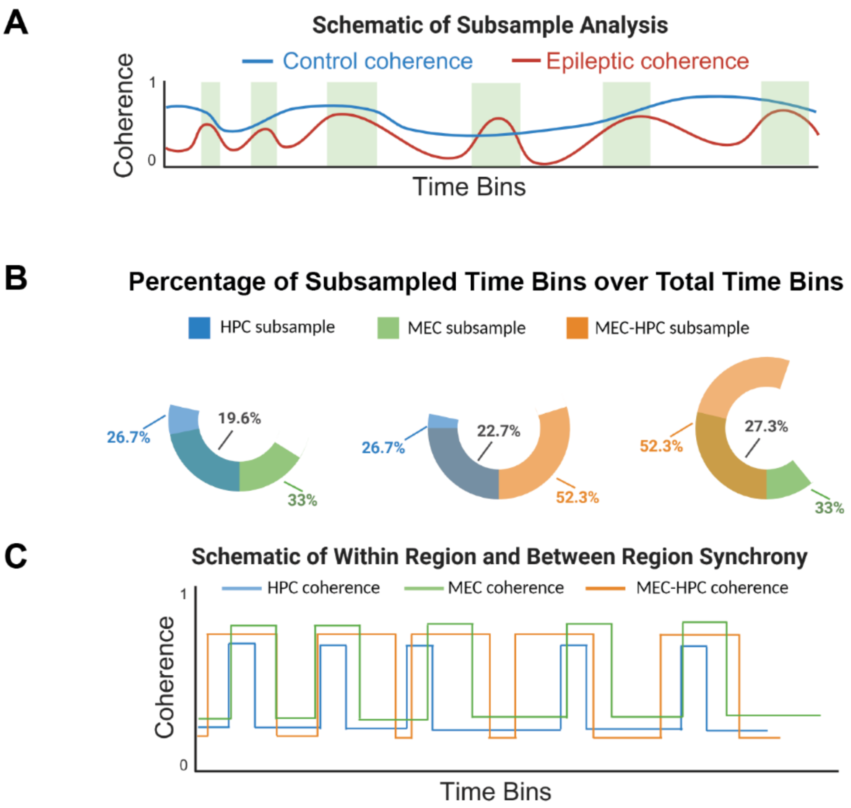

Supplementary Figure 7: Schematic of subsample analysis and relationship between subsampled time bins
A. Schematic of subsample analysis. Blue: coherence level in Control animals; Red: coherence level in Epileptic animals; Green: time periods that Epileptic animals have the same level of coherence as in the Control group. Periods in green were selected for subsample analysis.
B. Percent of time bins that were subsampled based on within-MEC, within-HPC, or MEC-HPC theta coherence, averaged across animals (HPC subsample: N = 11, 3wk Epileptic; N = 11, 8wk Epileptic; MEC subsample: N = 7, 3wk Epileptic; N = 5, 8wk Epileptic; MEC-HPC subsample: N = 10, 3wk Epileptic; N = 10, 8wk Epileptic). The percent of overlap in these time bins is shown in the darker shade.
C. Schematic of the within-region and across-region coherence profile. During the time HPC coherence (blue) is high, MEC-HPC (orange) coherence is also high; during the time MEC-HPC (orange) coherence is high, not all HPC (blue) or MEC (green) coherence are high.
